# Supplementary material for: Collaborative intelligence in AI: Evaluating the performance of a council of AIs on the USMLE
Source: PLOS Digit Health. 2025 Oct 9;4(10):e0000787. doi: 10.1371/journal.pdig.0000787 (PMC12510544; doi:10.1371/journal.pdig.0000787)
Supplement: S2 File — (ZIP) [file pdig.0000787.s002.zip › S2 Data Raw data files containing unprocessed question inputs and LLM outputs including transcripts of deliberations/STEP3_Question-30-1_Type-MC-NJ.docx]

**Facilitator** **[Round 1]:** A 68-year-old man comes to the office because of a 4-week history of increasing stiffness in his shoulders and upper arms. He is an avid golfer and has been unable to play in the morning because of the stiffness. He says that the stiffness is somewhat improved in the afternoon. He also has felt fatigued and sometimes ""headachy and feverish."" Medical history is significant for arthritis in his knees treated with acetaminophen, which has not relieved his shoulder and arm pain, and hyperlipidemia treated with pravastatin. He is a retired radiologist. Vital signs are temperature 37.7°C (99.8°F), pulse 76/min, respirations 18/min, and blood pressure 145/80 mm Hg. There is no tenderness in the shoulder girdle, and range of motion is normal bilaterally. The remainder of the physical examination discloses no abnormalities. Results of additional laboratorystudies are shown: Serum Blood Creatine kinase 88 U/L Hematocrit 36% Hemoglobin 12.0 g/dL WBC 8600/mm3 ESR 88 mm/h Which of the following is the most significant risk factor for the development of this patient's condition? (A) Age (B) Gender (C) History of osteoarthritis (D) Previous occupation (E) Use of pravastatin

**AI_B:** RESTATING THE QUESTION: The medical case presented is of a male patient, aged 68, exhibiting symptoms of increasing stiffness in his shoulders and upper arms, more prominent in mornings, coupled with fatigue, occasional headaches and a feverish sensation. His medical history records arthritis in the knees, which is currently ineffectively being managed with acetaminophen, and hyperlipidemia (a medical condition resulting in an excess of fats in the blood), managed with pravastatin. We must determine from the options provided, which factor is most significant in causing his present condition. PLANNING: Given the symptoms and test results, particularly the high erythrocyte sedimentation rate (ESR), a non-specific marker of systemic inflammation, one could hypothesize his condition is polymyalgia rheumatica (PMR). PMR commonly presents similar symptoms and high ESR in older individuals. The common risk factors for PMR include age, gender, genetic disposition, and environmental factors. Accordingly, we can assess the five choices. 1. Age: PMR affects people over 50, with the median onset age being 70. 2. Gender: PMR is more common in females. 3. History of osteoarthritis: Osteoarthritis is a wear-and-tear joint disease, unlikely to systemically influence the ESR. 4. Previous occupation: Unless his prior occupation was associated with systemic inflammation, it's highly unlikely to present a risk for PMR. 5. Use of pravastatin: The role of pravastatin, a cholesterol-lowering medication, as a risk factor in PMR is uncertain and needs further investigation. CLINICAL REASONING: Each risk factor has its merits, but we must prioritize the most substantial ones. While the patient's gender does play a role, it isn't the binding factor for PMR; both males and females can get it. Osteoarthritis history, his previous work, and the use of pravastatin don't carry enough substantiated weightage in primary PMR's pathophysiology. Therefore, the age of the patient warrants more scrutiny as it straddles the median onset age. CONCLUDING: Through process of elimination and identification of pertinent details concerning the patient's condition and comparing it with known risk factors, age is determined to be the most crucial factor in his present condition. RESPONSE: (A) Age is the most significant risk factor for the development of this patient's condition.

**AI_E:** RESTATING THE QUESTION: The patient is a 68-year-old man who is experiencing increased stiffness in his shoulders and upper arms that improves throughout the day. Other accompanying symptoms consist of fatigue, a headache, and feeling feverish. His medical history includes knee arthritis and hyperlipidemia for which he takes acetaminophen and pravastatin respectively. Interestingly, his clinical exam and basic labs are unremarkable except for an increased ESR (Erythrocyte Sedimentation Rate). Given these details, we are asked to identify the most significant risk factor that contributed to the development of this patient's condition. PLANNING: There are several assumptions we can identify, the first being the cause of the patient's symptoms. The patient's symptoms, in combination with an elevated ESR, suggest an inflammatory condition. We must also consider whether the symptoms are related to his age, gender, history of osteoarthritis, occupation, or the use of pravastatin as potential risk factors. Alternatively, we might question if an undiagnosed, underlying condition could be responsible. Given the symptomatology, the patient's age and high ESR, polymyalgia rheumatica (PMR) should be considered as one of the differential diagnoses. PMR, an inflammatory condition frequently seen in people over the age of 50, presents with morning stiffness, aching in the shoulder and hip girdle regions, constitutional symptoms like fatigue, and has a significant response to corticosteroids. ESR is typically elevated in these patients. Since this condition is more prevalent in older age groups, 'age' would be a highly relevant risk factor to consider. CLINICAL REASONING: Initiating from the assumptions highlighted above and the clinical presentation, we can start drawing connections. While all the options listed are risk factors for various health conditions, in light of the patient's symptoms, and laboratory findings, age seems the most compatible risk factor given the potential diagnosis of PMR. This patient's age puts him at a greater risk for this condition as it typically affects individuals aged 50 and older. It is also necessary to mention that the slight fever, elevated ESR, and feelings of malaise are manifestations of systemic inflammation, which is a characteristic of PMR. Furthermore, the particular stiffness pattern that improves as the day goes is also a classic PMR feature. CONCLUDING: Using a step-by-step clinical thought process, the condition that best matches the patient's clinical presentation and laboratory findings is polymyalgia rheumatica. This condition is most commonly seen in individuals who are 50 years of age or older, making 'age' the most significant risk factor. While the other options listed, such as gender, history of osteoarthritis, previous occupation, and use of pravastatin, do present certain health risk factors, they don't directly correlate with the patient's symptoms and potential diagnosis of PMR. RESPONSE: Given the age of the patient, the increasing stiffness in his shoulders and upper arms easing throughout the day, coupled with systemic symptoms like fatigue, headache, and elevated ESR levels, the diagnosis of PMR seems most likely. Consequently, the significant risk factor for this patient is his 'age' (A).

**AI_D:** RESTATING THE QUESTION: The query is seeking to determine the primary risk factor contributing to this 68-year-old man's current health issue, characterized by shoulder and arm stiffness that improves as the day progresses, as well as symptoms of fatigue, headache, and feverish feeling. A few important considerations in the patient's history include his age, gender, a prior diagnosis of osteoarthritis treated with acetaminophen, a history as a radiologist, and treatment with pravastatin for hyperlipidemia. The patient's laboratory results are also shown, including significantly elevated Erythrocyte Sedimentation Rate (ESR), a marker often used to indicate levels of inflammation in the body. PLANNING: If we consider the patient's symptoms, we can make a few assumptions. His symptoms, including increased stiffness in his shoulders and arms, primarily in the mornings, tiredness, and occasional headaches and fever, suggest an inflammatory process. The elevated ESR confirms the presence of inflammation. It's critical to determine which risk factor (age, gender, osteoarthritis, previous job, pravastatin use) is mainly contributing to his condition considering his clinical features. CLINICAL REASONING: Given the patient's history, we can associate these symptoms with an inflammatory condition rather than osteoarthritis, his previous occupation, or pravastatin use. Considering his age can also be an important factor, especially since many inflammatory diseases are more prevalent in older individuals. The primary suspect here is Polymyalgia Rheumatica (PMR), an inflammatory condition that predominantly affects older people. It is characterized by aching and morning stiffness in the shoulders, neck, and hip girdle areas. This fits the pattern of the patient's complaints and his particularly high ESR. PMR is more common in elders, predominantly in the age group of 50 or above. CONCLUDING: After considering all of the details provided about his case, it appears that his Age (option A on the list) is the most significant risk factor contributing to the patient's symptoms. The symptoms, general improvement in the afternoon, elevated ESR, and his age all align with a polymyalgia rheumatica diagnosis, a condition primarily seen in the elderly. RESPONSE: Therefore, in the face of the clinical scenario described, the usage of his age, specifically being over 50, is the most likely risk factor (A) for the development of this patient's condition, which likely is Polymyalgia Rheumatica. The age is contributing significantly more than the other options listed (Gender, History of Osteoarthritis, Previous Occupation, Use of Pravastatin) to the pathology of this patient's presentation.

**AI_C:** [RESTATING THE QUESTION] A 68-year-old man, a passionate golfer and retired radiologist, has been experiencing increasing morning stiffness in his shoulders and upper arms for the past 4 weeks. He also reports feeling fatigued, and sometimes experiences headaches and fevers. His medical history includes osteoarthritis in his knees, for which he takes acetaminophen, and hyperlipidemia, for which he is treated with pravastatin. His recent blood work shows slightly elevated temperature, mildly decreased hemoglobin and hematocrit levels, a normal WBC count, and significantly elevated ESR level. Surprisingly, there is no evident shoulder tenderness and his range of motion is normal. This question requires identifying the most significant risk factor for his current condition out of the following: His age, gender, history of osteoarthritis, past work as a radiologist, or use of pravastatin as a medication. [PLANNING] It is important to consider each possible risk factor. Age could be a factor as many conditions develop with age. Being male could influence certain diseases such as those related to prostate (which is unlikely here). History of osteoarthritis could contribute, but typically this condition is limited to painful joints, not shoulder stiffness specifically or general feelings of malaise. His past job as a radiologist could be a factor if his condition is related to radiation exposure. Use of pravastatin could be related if the condition is a side effect of this drug. Despite all these possibilities, the elevated ESR (erythrocyte sedimentation rate) stands out. ESR is an inflammatory marker; its significant elevation often indicates ongoing systemic inflammatory process. Furthermore, his symptoms of morning stiffness that improve as the day progresses, along with a general malaise, suggest polymyalgia rheumatica (PMR), a condition more common in older adults. [CLINICAL REASONING] Polymyalgia rheumatica is an inflammatory disease that causes muscle pain and stiffness, especially in the shoulders and hips. Symptoms often get worse in the mornings. It is more common in individuals over 50 (correlates with this patient's age), and while gender does influence the prevalence ratio (slightly more common in women), it is not as strong a risk factor as age. Osteoarthritis is not a predisposing factor for PMR, and while radiologists may have higher incidences of certain malignancies or degenerative diseases due to radiation exposure, there is no evidence pointing to an increased risk for PMR. Finally, pravastatin, a statin drug used for managing hyperlipidemia, does not increase the risk for PMR, although muscle stiffness and pain may occur as side effects. However, these side effects would likely be accompanied by an elevation in creatine kinase levels, which is not reported in this patient's labs. Therefore, out of all risk factors, age is the most significant one for the suspected condition, polymyalgia rheumatica. [CONCLUDING] In analyzing the patient's symptoms of morning stiffness, especially in the shoulders, general malaise, and an elevated ESR, alongside other possible risk factors and disease correlations, it appears that the most significant risk factor for the development of this patient's condition, suspected to be polymyalgia rheumatica (PMR), is his age. [RESPONSE] (A) Age is the most significant risk factor for the development of this patient's condition. The condition likely is Polymyalgia Rheumatica, an inflammatory condition more common in adults over age 50.

**AI_A:** RESTATING THE QUESTION: The question inquires about the primary risk factors that could have contributed to this 68-year-old man's apparent medical condition. He has been dealing with stiffness in his shoulders and upper arms along with fatigue and occasional feelings of being feverish and headachy. This stiffness is more pronounced in the mornings and eases up somewhat in the later part of the day. His medical history includes arthritis in the knees and hyperlipidemia. He takes acetaminophen and pravastatin respectively for the conditions. The patient formerly had a career in radiology. Although he presents with minor feverish symptoms, there is no soreness in the shoulder girdle and his shoulder movements are not restrained. Among the things needing a closer look would be the man's advanced age, male gender, history of osteoarthritis, past occupation, and his pravastatin use. PLANNING: Looking at the facts available, we can make several assumptions. First, his age and gender may put him at a higher risk for certain conditions, but without additional evidence, it is difficult to definitively link these factors to his current symptoms. Second, his history of osteoarthritis could be contributing to his symptoms, but given that the pain in his shoulders and arms is not alleviated by acetaminophen, which is used to treat the arthritis in his knees, a link to his current symptoms is less likely. Lastly, both his previous occupation as a radiologist and usage of pravastatin need to be considered, as they could potentially contribute to his current symptoms. CLINICAL REASONING: The patient's symptoms, along with the elevated ESR, suggest the possibility of an inflammatory process. Osteoarthritis can lead to morning stiffness, however, it's mainly associated with wear and tear, and does not typically result in systemic symptoms like fatigue, fever, or an elevated ESR, so it’s less likely to contribute significantly to this case. His age and gender are general risk factors for many conditions but without further tying information, it can’t be clearly associated with the current scenario. His previous occupation as a radiologist would likely expose him to extended periods of radiation, but without signs of radiation-related damages present, this is also unlikely to be significant in this context. Lastly, pravastatin, a statin used for treating hyperlipidemia, has been associated with side effects such as muscle pain and stiffness, and in rare cases, a condition called statin-induced polymyalgia rheumatica (PMR). However, causality is not clear-cut but is a possibility that requires consideration, given the correlation of symptoms. CONCLUDING: Considering all these facts and making clinical associations, while the link between statin use and PMR isn't entirely clear-cut, the presence of systemic symptoms, and the marked relief in pain as the day goes on understandably puts the focus on PMR. Even though the statin, pravastatin, is not definitively proven to cause PMR, it's the variable that holds the more significant possibility in this case. RESPONSE: The most significant risk factor for the development of this patient's condition is (E) Use of pravastatin.

**Facilitator** **[Round 2]:** Summary: AI_A believes that the most significant risk factor for the patient's condition is the use of pravastatin. They argue that the patient's symptoms and elevated ESR suggest an inflammatory process, which could be linked to pravastatin use, as it has been associated with muscle pain and stiffness, and in rare cases, a condition called statin-induced polymyalgia rheumatica (PMR). AI_B, AI_C, AI_D, and AI_E all agree that the patient's age is the most significant risk factor. They all suggest that the patient's symptoms and elevated ESR are indicative of polymyalgia rheumatica (PMR), a condition more common in older adults. They argue that the patient's age is a stronger risk factor than his gender, history of osteoarthritis, previous occupation, or use of pravastatin. Key Differences: The key difference between the perspectives lies in the interpretation of the patient's symptoms and the potential link to pravastatin use. AI_A believes that the patient's symptoms could be a side effect of pravastatin use, while AI_B, AI_C, AI_D, and AI_E believe that the patient's symptoms are more indicative of PMR, a condition more common in older adults. Question for Council Members: Could you provide more insight into the potential link between pravastatin use and the patient's symptoms? How does the risk associated with pravastatin use compare to the risk associated with the patient's age in the development of PMR?. Based on your preceding response, how would you answer the original question: [A 68-year-old man comes to the office because of a 4-week history of increasing stiffness in his shoulders and upper arms. He is an avid golfer and has been unable to play in the morning because of the stiffness. He says that the stiffness is somewhat improved in the afternoon. He also has felt fatigued and sometimes ""headachy and feverish."" Medical history is significant for arthritis in his knees treated with acetaminophen, which has not relieved his shoulder and arm pain, and hyperlipidemia treated with pravastatin. He is a retired radiologist. Vital signs are temperature 37.7°C (99.8°F), pulse 76/min, respirations 18/min, and blood pressure 145/80 mm Hg. There is no tenderness in the shoulder girdle, and range of motion is normal bilaterally. The remainder of the physical examination discloses no abnormalities. Results of additional laboratorystudies are shown: Serum Blood Creatine kinase 88 U/L Hematocrit 36% Hemoglobin 12.0 g/dL WBC 8600/mm3 ESR 88 mm/h Which of the following is the most significant risk factor for the development of this patient's condition? (A) Age (B) Gender (C) History of osteoarthritis (D) Previous occupation (E) Use of pravastatin]

**AI_D:** Restatement: The inquiry wishes to determine the most significant risk factor contributing to the development of the symptoms of a 68-year-old patient: notably stiffness in his shoulders and upper arms that's more pronounced in the mornings, fatigue, occasional headaches, and feverish symptoms, along with a significantly elevated ESR. The condition seems not to be linked to his previously diagnosed osteoarthritis or mediated by the patient's acetaminophen use. Other past medical history includes hyperlipidemia managed with pravastatin. Given his set of symptoms, clinical history, and laboratory findings, we are tasked with ascertaining the biggest risk factor. Planning: Several factors serve as potential variables for the patient’s condition. We could hypothesize a possible impact of his age (68 years), gender, history of osteoarthritis, his prior profession as a radiologist, or his use of pravastatin. Given his high ESR and constellation of symptoms that suggest systemic inflammation, determination of the leading risk factor is essential. Clinical Reasoning: Research suggests that pravastatin, like many statins, has been linked to muscle-related side effects, which could explain the patient's symptoms. However, his laboratory results show a normal Creatine kinase level which contradicts this idea. Comparatively, his apparent symptomatology aligns considerably with Polymyalgia Rheumatica (PMR), an inflammatory condition characterized by muscle pain and stiffness in the neck, shoulders, and hip area. Typically, PMR affects adults above the age of 50 and is more prevalent as people age. Therefore, despite the possible influence of other factors, it is likely that the age factor offers a more comprehensive explanation for the onset of his condition. Concluding: Taking this into consideration, the patient's age, coupled with his symptoms and elevated ESR index, suggests the most significant risk factor for the development of his condition could be age, given that his symptomatology aligns strongly with Polymyalgia Rheumatica (PMR). Response: Thus, in answering the question relating to the 68-year-old patient’s condition, (A) Age is likely the most significant risk factor for the development of this patient's condition.

**AI_B:** If we were to look into your question further, we need to address two focuses: the potential connection between pravastatin and this patient's symptoms and how the associated risks compare with the risk his age presents in the development of Polymyalgia Rheumatica (PMR), the likely diagnosis. Pravastatin, a cholesterol-lowering medication, has been associated with muscle pain and stiffness, suggesting a possible link to PMR symptoms. Some research reports have noted a possible risk of PMR with statin use. However, this is considered relatively rare and not as well-established as the risk associating age with PMR. Polymyalgia Rheumatica (PMR) is an inflammatory condition causing pain and stiffness in the shoulder and hip area. It's more common in individuals aged 50 years and above, with most patients diagnosed in their 70's. This makes age a significant risk factor. The patient's symptoms, including morning stiffness that improves later in the day, are classic symptoms of PMR. Comparing both, though pravastatin use may have contributed to the patient's symptoms, the unrestricted risk attributed to age seems to exceed that of pravastatin use based on prevalence data and symptom presentation. Given this evidence, in answering the initial question, "Which of the following is the most significant risk factor for the development of this patient's condition?", it seems safe to choose "(A) Age" as the most significant risk factor for the development of PMR in this patient. However, a cautious approach warrants further investigation of the potential role of pravastatin in contributing to these symptoms. This should include monitoring the symptom response to pravastatin discontinuation and considering referral for rheumatological evaluation if symptoms persist. This patient's management should be tailored to balance the risk-benefit ratio of continuing the pravastatin, given the utility in managing his hyperlipidemia.

**AI_C:** Restating the question: This patient is displaying symptoms such as shoulder and arm stiffness that improves throughout the day, fatigue, occasional headaches, and fevers. He has a medical history of arthritis and hyperlipidemia, for which he takes acetaminophen and pravastatin respectively. Despite a lack of physical abnormalities in shoulder movement, his lab results show an elevated erythrocyte sedimentation rate (ESR). The question is what contributes most significantly to his current condition among age, gender, a history of osteoarthritis, previous occupation, or the use of pravastatin. Planning: Considering his symptoms of morning stiffness and raised ESR, conditions like polymyalgia rheumatica come to mind, which are associated with age. But it's important not to ignore the patient's use of pravastatin, a statin that can lead to muscle-related side effects. However, the normal creatine kinase levels argue against statin-induced myalgia. Our task is to clinically prioritize which amongst these factors - age, gender, osteoarthritis history, previous occupation, or pravastatin use - is most significant in his current condition. Clinical Reasoning: As mentioned earlier, morning stiffness and an elevated ESR can be indicative of an inflammatory condition like polymyalgia rheumatica (PMR), which is predominantly seen in people over 50. This patient at 68 should be evaluated with this in mind. PMR does not affect the range of motion, which aligns with our patient's situation, and other symptoms like fatigue, headaches, and fever can also be seen in PMR. However, we must consider pravastatin, the lipid management drug he has been taking, which has muscle pain and stiffness as potential side effects, though more often leading to increased CK levels, not seen in the patient. His gender, previous work as a radiologist and history of osteoarthritis do not highly support our initial assumption of PMR or any associated condition. Concluding: The main focus of suspicion in this patient's condition seems to revolve around an inflammatory condition likely to PMR based on his age, symptoms, and lab results. His gender, previous occupation, history of osteoarthritis, and pravastatin use do not significantly contribute to a more likely diagnosis compared to age being a prime risk factor for PMR. Response: Based on the clinical thinking process so far, the most significant risk factor for the development of this patient's condition seems to be (A) Age.

**AI_A:** (1) RESTATING THE QUESTION: Our task is to identify what the significant risk factor is behind the patient's symptoms, which include stiffness in the shoulders and upper arms, fatigue, and feeling feverish occasionally. The symptoms depict musculoskeletal and systemic discomfort that seem to improve as the day progresses. The abnormal laboratory finding is an elevated Erythrocyte Sedimentation Rate (ESR), which is a marker of inflammation in the body, indicates an underlying systemic issue. (2) PLANNING: Several assumptions can be made in light of the symptoms and findings. Given the elevated ESR, the symptoms could be due to an underlying systemic inflammatory condition. Other risk factors to consider are age, gender, history of osteoarthritis, previous occupation, and use of pravastatin. An alternate assumption would be to suspect that the condition might be drug-induced, considering that the patient is on pravastatin and this drug has been associated with muscle-related symptoms. The assumption that should be prioritized is evaluating whether the patient's symptoms align more with polymyalgia rheumatica (PMR), an inflammatory condition more common in older adults, or a possible side effect of pravastatin. (3) CLINICAL REASONING: PMR typically affects people above the age of 50 and is characterized by stiffness in the neck, shoulders, and hip area that is worse in the morning and improves as the day progresses. The arthritic pain in our patient's upper arms and shoulders is consistent with this. ESR is notably raised in PMR, correlating with our patient's lab results. The patient is also 68 years old, which correlates with the patient profile associated with PMR. Statin-induced muscle symptoms are also a possibility as muscle aches and pains are common side effects of statin therapy, including pravastatin. However, the patient's symptoms, combined with the elevated ESR, lean more towards PMR than statin-induced side effects, which often present with muscle weakness and an elevation in creatine kinase levels, which is not the case here. (4) CONCLUDING: The patient's age, symptoms, and elevated ESR all point towards a probable diagnosis of PMR, which is typically associated with older adults over the age of 50. (5) RESPONSE: Given the above reasoning, the patient's age (A) is the most significant risk factor for the development of their condition.

**AI_E:** Restating the question: The patient is a 68-year-old man who has been suffering from increasing stiffness in his shoulders and arms, along with associated symptoms such as fatigue, headaches, and feverish feelings. Despite having no physical abnormalities, the patient has an elevated erythrocyte sedimentation rate (ESR), a marker usually linked to inflammation or infection. The question seeks to identify the most significant risk factor associated with the patient's condition. Understanding this would perhaps help to plan targeted therapeutic strategies and provide better care to the patient. Now, let's consider the possible assumptions. The potential risk factors provided are – age, gender, history of osteoarthritis, previous occupation, and use of pravastatin. Age could be a significant risk factor, as some health complications tend to increase with age. Gender too can be a determinant for certain conditions. A previous history of osteoarthritis may suggest susceptibility to joint-related issues. The patient's previous occupation in the radiology field could have exposed him to risk factors related to his workplace environment. Finally, the use of pravastatin is also a potential risk factor as some drugs might have adverse side effects, including muscle stiffness or discomfort. Upon evaluating these potential assumptions, it seems plausible that the most clinically significant risk factor to prioritize would be age. Polymyalgia rheumatica is a clinical syndrome characterized by severe aching and stiffness in the muscles around the neck, shoulder girdle, and hip girdle, particularly in individuals above 50 years of age. The patient's elevated ESR is also consistent with this condition. However, close consideration of the potential side effects of pravastatin may also be warranted. Through clinical reasoning, it's noted that polymyalgia rheumatica (PMR) is highly suspected in this case due to the patient's age, symptomatology, and elevated ESR. PMR is much more common in individuals aged over 50, with the average age at diagnosis being 70. The patient's symptoms, including morning stiffness that improves during the day and systemic symptoms such as fatigue, align with this diagnosis. Although some symptoms can mimic those seen with statin use, like pravastatin, the absence of muscle tenderness, normal creatine kinase levels, and the elevated ESR suggest an inflammatory condition like PMR rather than a drug side effect. Concluding, although certain assumptions warrant consideration, available evidence primarily leans towards the patient's age as the most significant risk factor. While pravastatin use can cause certain similar symptoms, our patient's age, symptoms, and clinical findings align more closely with the characteristics of polymyalgia rheumatica, a condition often found in older adults and rarely associated with statin use. Finally, based on the outlined reasoning, my answer to the question - "Which of the following is the most significant risk factor for the development of this patient's condition?" would be (A) Age.

**Facilitator** The consensus among the AI responses is that the most significant risk factor for the development of this patient's condition is (A) Age. The patient's symptoms, including stiffness in the shoulders and upper arms that improves throughout the day, fatigue, occasional headaches, and feverish feelings, along with an elevated Erythrocyte Sedimentation Rate (ESR), suggest an underlying systemic inflammatory condition. These symptoms align closely with Polymyalgia Rheumatica (PMR), an inflammatory condition more common in individuals aged 50 years and above. While the patient's use of pravastatin, a cholesterol-lowering medication, could potentially contribute to muscle-related symptoms, the normal Creatine kinase levels and the elevated ESR suggest an inflammatory condition like PMR rather than a drug side effect. Furthermore, the risk associated with age in the development of PMR is more well-established and prevalent than the risk associated with statin use. Therefore, based on the patient's age, symptoms, and laboratory findings, age is identified as the most significant risk factor for the development of his condition. However, a cautious approach would warrant further investigation into the potential role of pravastatin in contributing to these symptoms, including monitoring the symptom response to pravastatin discontinuation and considering referral for rheumatological evaluation if symptoms persist.
